# Learning about stress from building, drilling and flying: a scoping review on team performance and stress in non-medical fields

**DOI:** 10.1186/s13049-021-00865-7

**Published:** 2021-03-25

**Authors:** Femke S. Dijkstra, Peter G. Renden, Martijn Meeter, Linda J. Schoonmade, Ralf Krage, Hans van Schuppen, Anne de la Croix

**Affiliations:** 1 Department of Educational Sciences, Vrije Universiteit Amsterdam, Van der Boechorststraat 7, Amsterdam, the Netherlands; 2 Academy of Health Sciences, Saxion University of Applied Sciences, Handelskade 75, Deventer, the Netherlands; 3 Faculty of Health, Nutritrion and Sport, The Hague University of Applied Sciences, Johanna Westerdijkplein 75, The Hague, the Netherlands; 4 Department of Human Movement Sciences, Vrije Universiteit Amsterdam, Van der Boechorststraat 7, Amsterdam, the Netherlands; 5 Medical library, Vrije Universiteit Amsterdam, De Boelelaan 1117, Amsterdam, the Netherlands; 6 Department of Anesthesiology, KJF Klinik St. Elisabeth, Müller-Gnadenegg-Weg 4, Neuburg an der Donau, Germany; 7 Department of Anesthesiology, Amsterdam UMC, University of Amsterdam, Meibergdreef 9, Amsterdam, the Netherlands; 8 Research in Education, Amsterdam UMC, Vrije Universiteit Amsterdam, De Boelelaan 1117, Amsterdam, the Netherlands

**Keywords:** Teams, Stress, Team performance, Performance psychology, Critical care, Emergency care, Crew resource management, Human factors

## Abstract

**Background:**

Teamwork is essential in healthcare, but team performance tends to deteriorate in stressful situations. Further development of training and education for healthcare teams requires a more complete understanding of team performance in stressful situations. We wanted to learn from others, by looking beyond the field of medicine, aiming to learn about a) sources of stress, b) effects of stress on team performance and c) concepts on dealing with stress.

**Methods:**

A scoping literature review was undertaken. The three largest interdisciplinary databases outside of healthcare, Scopus, Web of Science and PsycINFO, were searched for articles published in English between 2008 and 2020. Eligible articles focused on team performance in stressful situations with outcome measures at a team level. Studies were selected, and data were extracted and analysed by at least two researchers.

**Results:**

In total, 15 articles were included in the review (4 non-comparative, 6 multi- or mixed methods, 5 experimental studies). Three sources of stress were identified: performance pressure, role pressure and time pressure. Potential effects of stress on the team were: a narrow focus on task execution, unclear responsibilities within the team and diminished understanding of the situation. Communication, shared knowledge and situational awareness were identified as potentially helpful team processes. Cross training was suggested as a promising intervention to develop a shared mental model within a team.

**Conclusion:**

Stress can have a significant impact on team performance. Developing strategies to prevent and manage stress and its impact has the potential to significantly increase performance of teams in stressful situations. Further research into the development and use of team cognition in stress in healthcare teams is needed, in order to be able to integrate this ‘team brain’ in training and education with the specific goal of preparing professionals for team performance in stressful situations.

**Supplementary Information:**

The online version contains supplementary material available at 10.1186/s13049-021-00865-7.

## Introduction

Healthcare professionals, especially in emergency and critical care, regularly find themselves in situations in which they have to treat patients, with colleagues from different disciplines, under different types of pressure [1], for example during cardiopulmonary resuscitation (CPR) or trauma resuscitation. Although work routines for situations such as CPR are highly standardized [2], teams have to be able to react to unexpected events. It is precisely during those unexpected events that the quality of non-technical skills (e.g. leadership, communication, teamwork) plays a decisive role for patient safety [3]. For instance, Krage et al. [4] showed that during CPR with external distractors (e.g. noise or presence of family members), the quality of non-technical skills decreased, due to the influence of stress during these situations. Hence, they argued that medical teams should adopt interventions that improve the quality of non-technical skills in stressful situations [4]. A significant factor in these situations is *stress*: stress can be described as an imbalance between situational demands and personal resources, and can be either beneficial or destructive to performance [5, 6]. Thus far, many studies have shown that performance often deteriorates under stress or high pressure [7–10]. Since stress is unavoidable in healthcare, even when preventative measures are taken, patient care might benefit from team performance improvement strategies.

Research on teams within healthcare has looked into the relevant competences for teamwork in order to improve patient safety [11, 12]. Most of this research concentrates on the acute care setting and demonstrates there is room for improvement concerning team performance in stressful situations. Ranging from the potential role of psychological skills in order to reduce the effect of stress, to the impact of certain training methodologies on stress levels within teams, multiple studies show a specific interest in stress [4, 13–16]. However, the essentials for team performance in stressful situations remain somewhat unclear and require further investigation.

This might also apply for the different training methodologies that have increasingly been used in certain fields within healthcare, for example crew resource management (CRM) and simulation training [17]. An important similarity in these training methodologies is the focus on non-technical skills, such as communication, shared understanding and situational awareness [18]. Different reviews show a tremendous amount of knowledge on these specific training methodologies [17, 19, 20]. Yet, specific requirements for team performance in stressful situations appear to be uncharted territory.

We believe that inspiration from fields outside of medicine can help with further development of training and education for health care teams, by increasing understanding of team performance in stressful situations. In a broader context, this has already led to improved quality and safety of healthcare [21, 22]. However, in order to create a more profound understanding of stress, the potential effects of stress and specifically the relationship with team performance, it could be worthwhile to explore the scope of knowledge and good examples outside the field of healthcare once again. We hope to unlock insights on team performance and stressful situations.

In this review we take a careful and systematic look beyond healthcare to learn about teams, stress and performance. We aim to provide a comprehensive overview of a) sources of stress (*what is the cause?*), b) effects of stress on teams (*what happens?*) and c) concepts on dealing with stress (*what helps?*). This may confirm already existing ideas or could suggest new insights into how to study, teach and train teams in healthcare and ultimately improve patient safety.

## Methods

We used a scoping review methodology [23] for a broad analysis of the concept of stress among teams in disciplines outside of healthcare. The scoping review methodology systematically surveys an area of research within all types of studies, with the focus on identification and/or clarification of important factors related to a certain concept [24]. The five steps described by Arksey and O’Malley for conducting a scoping review [25, 26] were used in combination with the Preferred Reporting Items for Systematic Reviews and Meta-analyses (PRISMA) and the PRISMA-ScR (scoping review) statement [27, 28].

Scoping reviews are not eligible for registration in the Prospero-database [29].

### Literature search and study design

In order to cover the broadest range of interdisciplinary literature, best fitting to our research question, we searched the following bibliographic databases Scopus, Web of Science Core Collection and PsycINFO (via Ebsco), selecting articles published in English between January 1st 2008 and October 27th 2020, in collaboration with a medical librarian (*LS*). Sources of evidence that were considered for inclusion were articles, articles in press, and reviews.

Since our pilot search with the terms ‘stress’, ‘team’ and ‘performance’ already returned many results, we chose to restrict ourselves to a date limit of 10 years (2008–2018, update search in 2020). Search terms included controlled terms (Thesaurus terms in PsycINFO), as well as free text terms. The following terms were used (including synonyms and closely related words) as index terms or free-text words: ‘team’, ‘stress’, ‘coping’ and ‘performance’. The full search strategies for all databases can be found in Supplementary File 1.

### Study selection

A first selection of retrieved articles was done based on title alone (by *PR and FD*), in order to exclude studies relating to non-humans, or stress-related physical or psychological disorders, for example. Abstracts were independently reviewed (by *AC and FD*) using Covidence, possible discrepancies were discussed and consensus was reached.

For inclusion in the last step, review of full-text, the article needed to:

- Focus on teams working together in realistic stressful or high-pressure situations;
- Focus on the effects of stress on team performance, or focus on the appraisal of and coping with stress;
- Include the outcome measure: experienced stress or pressure by the team

A complete overview of the inclusion / exclusion criteria throughout the steps mentioned above can be found in Supplementary File 2. Every step in the screening process was done by at least two members of the interprofessional research team and discussed within the full team.

### Data-extraction & analysis

The data extraction form was designed on the basis of the research questions. After a pilot session some adaptations were made, which resulted in the final data extraction form that can be found in Supplementary File 3.

Analysis started with the facts from the data charting form. Frequent recurring themes such as shared mental model were highlighted and discussed within the team. In order to create more focus the in-depth analysis addressed potential leads for educational interventions.

The qualitative content analysis was an iterative process, in which the team often discussed topics found in the data. A final synthesis of the data was done by two researchers (by *AC and FD*), resulting in the three main themes as seen in the results: conceptualizing stressors (what is the cause?), effect of stress on team performance (what happens?), and helpful team processes in stressful situations (what helps?) [28, 30].

We performed a critical appraisal of the studies based on a general checklist by Hawker et al. [31]

## Results

**Fig. 1:**
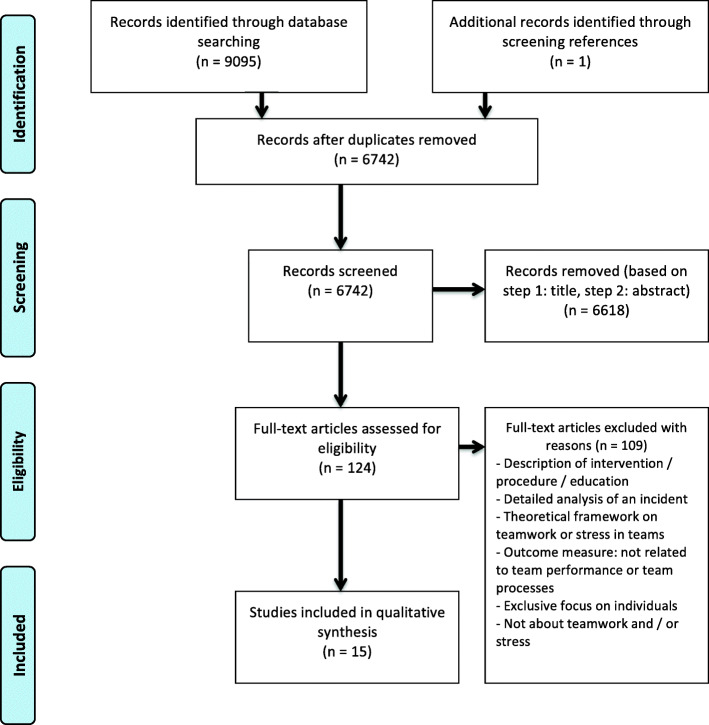
Flow diagram of search, based on PRISMA Statement

The full search (see Fig. 1) yielded 9095 studies (including duplicates), with topics ranging from papers about stress in pineapple trees to the potential threat of nuclear weapons. After excluding irrelevant studies following the screening process described in the Methods section, 15 articles were identified to be included in the final analysis.

Table 1 shows descriptive characteristics of the studies included (partially based on NICE-algorithm for classifying quantitative study designs [32] and Hawker et al. [31]); Table 2 shows the specifics on stress, teams, performance and training.

**Table 1:** Characteristics of included studies in scoping review

Study	Study design [32]	Discipline	Sample size	Study location	Quality of evidence [31]
Gardner, 2012 [33]	Non-comparative study – multi method	Accounting & consultancy	Study 1: 78 teams Study 2: 6 teams	United States of America	Fair
Savelsbergh et al., 2012 [34]	Non-comparative study	Building & construction	38 teams	The Netherlands	Good
Gervits et al., 2016 [35]	Non-comparative study – mixed methods	Student teams	10 teams	United States of America	Fair
Long et al., 2014 [36]	Non-comparative study	Student teams	55 teams	China	Poor
Bourgeon et al., 2013 [37]	Non-comparative study – multi method	Aviation	10 teams	France	Poor
Ellis et al., 2011 [38]	Cluster-RCT	Student teams	54 teams	United States of America	Fair
Kaplan et al., 2013 [39]	Non-comparative study – multi-method	Nuclear engineering	21 teams	United States of America	Fair
Pearsall et al., 2009 [40]	Cluster-RCT	Student teams	83 teams	United States of America	Fair
Price et al., 2017 [41]	Cross-sectional study	Army	18 teams	United States of America	Poor
Stachowski et al., 2009 [42]	Non-comparative study – multi-method	Nuclear engineering	14 teams	United States of America	Fair
Maruping et al., 2015 [43]	Non-comparative study	Software firm	111 teams	United States of America	Fair
Espevik et al., 2013 [44]	Non-comparative study	Navy	Not applicable	Norway	Poor
Espevik et al., 2011 [45]	Non-RCT	Navy	23 teams	Norway	Good
Xu et al., 2018 [46]	Before-and-after or interrupted time series	Student teams	36 teams	United States of America	Good
Wang et al., 2020 [47]	Non-comparative study – multi method	Nuclear engineering	Study 1: 18 individuals Study 2: 5 teams	China	Good

**Table 2:** Specifics on stress, teams, performance and training

Study	Conceptualizing stress	Effect of stress in teams	Strategies to deal with stress	Training possibilities
Gardner, 2012 [33]	Performance pressure	Focus on general expertise.	Planning, knowledge coordination and morale-building communication. Presence, recognition and acknowledgement of domain-specific expertise.	Learn to recognize specific knowledge.
Savelsbergh et al., 2012 [34]	Role pressure	Inhibition of team learning behaviours. Focus on primary task processes.	Sharing experiences, collective reflection and feedback.	Time for team learning: collective sense making of problems.
Gervits et al., 2016 [35]	Time pressure	Increase of disfluency rate and speech rate. Fewer self-repairs.	Grounding within the team (seeking information, engaging, monitoring)	No specific training strategies mentioned in study.
Long et al., 2014 [36]	Time pressure	Increase of task conflicts due to divergent views.	Formation of team mental model (prevention of conflict and divergent views)	No specific training strategies mentioned in study.
Bourgeon et al., 2013 [37]	Crisis: unexpected and ambiguous situation	Rigid behaviour, due to lack of cognitive flexibility.	Achievement of shared representation of the situation.	Development of training specifically focused on cognitive flexibility through communication.
Ellis et al., 2011 [38]	Time pressure	Lower levels of accuracy of mental models and less information allocation.	Increase in accuracy of mental model and decrease in tension after cross training.	Cross training in order to learn about roles and responsibilities of teammates.
Kaplan et al., 2013 [39]	Time pressure	Development of negative emotions.	Reduction negative emotions through a certain base level of positive affect within the team.	Considerations about team composition based on the affective dispositions of team members. Training not possible.
Pearsall et al., 2009 [40]	Time pressure and role pressure	In presence of avoidant coping strategy: lower levels of transactive memory and more psychological withdrawal.	Use of problem solving coping strategy to improve team performance and reach higher levels of transactive memory.	Learning to adopt problem solving coping strategy.
Price et al., 2017 [41]	Performance pressure, through affective stressors	Reduction of objective situational awareness (SA). Possible overconfidence in SA.	Experience of team engagement results in less overconfidence in SA and less risk-taking.	Foster perception of team engagement.
Stachowski et al., 2009 [42]	Time pressure and crisis	More information requests within team, and less information transfers.	Best performance in stress by teams with fewer, shorter, less complex and more flexible interaction patterns.	Training to foster brief team interaction patterns, without sacrificing shared team knowledge.
Maruping et al., 2015 [43]	Time pressure and performance pressure	Narrow focus on task execution and withdrawal from task management.	Improvement of processes like planning, evaluation and monitoring in the presence of strong team leadership.	Training of processes like planning, evaluation and monitoring progress.
Espevik et al., 2013 [44]	Time pressure	Influences coordination and cooperation in teams in absence of team familiarity	Knowledge about team members.	Potential effects of cross training are discussed.
Espevik et al., 2011 [45]	Crisis	In absence of team familiarity less adaptive behaviour and less closed-loop communication.	Familiarity between team members.	Training with unfamiliar team members (cross training)
Xu et al., 2018 [46]	Task stress (i.e. performance pressure) and technological stress	Lower levels of positive affect (PA) and increase in reaction time.	Higher mean levels of PA due to positional rotation training.	Positional rotation training (variant of cross training) to enhance shared mental model.
Wang et al., 2020 [47]	Time pressure and crisis	Narrowing of scope and less understanding of others’ operational status	Best performance in stress by teams showing adaptive workload management, and proactive performance monitoring.	Teamwork training to develop and strengthen coordination and adaptation. Recognition of behaviour patterns of team members.

### Conceptualizing stressors – what is the cause?

In the fifteen papers, a myriad of terms was used for stress, often inconsistently, among which mental stress [35], tension [38], and crisis [37, 39, 42, 44, 47]. These terms all referred to situations or feelings in which demands exceeded the resources of the individual or the team. The sources of stress found in the data could be categorized as at least three non-mutually exclusive types: 1) performance pressure; 2) role pressure; and 3) time pressure.

#### Performance pressure

Teams can feel a shared responsibility for results, accompanied by close examination and evaluation by supervisors and clients, and associated consequences (such as a financial bonus) of the end-result of a project [33, 41, 43, 46]. This type of pressure can be perceived as an external force imposed on the team.

#### Role pressure

A team of students with no awareness of their team members’ expertise was at risk of experiencing role pressure [38]. Role pressure could be described as doubt in team members about their own role and/or the role of others, due to ambiguity, conflict or overload in task requirements [34, 36, 38, 40].

#### Time pressure

This type of pressure was presented as an external demand for the team to accomplish tasks within a fixed time period [35–37, 39, 42–44, 47]. Although time is an objective measure, team members can perceive time pressure differently. This subjectivity relates to why time pressure was experienced as a challenge stressor as well [40].

Terms like ‘uncertainty’, ‘challenge’ and ‘threat’ were described as the resulting feeling within teams when they experienced one of the stressors mentioned before. *Uncertainty* related to roles [34, 38, 40, 44] or the situation [37, 39, 44, 47]. *Threat* was the perception that some element in the environment might cause harm to the individual [38, 40]. When perceiving a situation as a *challenge*, individuals and teams felt more motivated to fulfil the job [40].

### Effect of stress on team performance – what happens?

Teams experiencing stress reportedly felt the effects in numerous ways, with impairing effects predominating in the included studies. A narrow focus, unclear responsibilities, diminished understanding of the situation and emotional effects were considered to be the major consequences of working in a stressful environment.

#### A narrow focus

Several teams exhibited a specific focus on task execution, which moved their attention away from secondary team processes like coordination, planning and team learning [43]. Members from nuclear power plant crews acknowledged this narrowing focus as a potential cause for coordination breakdowns as well [47]. Especially in stressful circumstances like simultaneous technical failures during a flight, a tendency towards task execution alone resulted in more rigid behaviour of a team and a potential decline in team performance [37].

#### Unclear responsibilities

In order to be flexible as a team, expertise of all team members should be known, acknowledged, and used at the right moment [33, 40]. Due to not knowing who knows what, and who is responsible for what a decline in team performance was observed in student teams [36, 38, 46] and navy teams [44, 45] and mentioned by members of nuclear engineering teams [47].

#### Diminished understanding of the situation

Due to inaccuracies in understanding between team members, the way information was distributed within a team could change [37, 41, 42]. A decrease in situational understanding was explicitly seen in research on action teams composed of students who were not previously acquainted with each other [38]. Without receiving necessary information for certain actions, team members were not able to anticipate what was needed in that particular situation [38].

#### Emotional effects

The average level of emotions in nuclear power plant teams appeared to impact the performance of a team in a stressful setting [39, 46]. Presence of a wide array of emotions was negatively related to the awareness of what was happening around the teams, the management of their cognitive resources, the exchange of information and their collaboration [39].

### Helpful team processes in stressful situations - what helps?

Helpful team processes were observed to maintain or even improve team performance in stressful situations. Three main topics retrieved from the included articles are *communication*, *shared understanding and knowledge*, and *situational awareness*.

#### Communication

In terms of building morale [33], familiarizing with the team [35], preventing conflicts [36] and regulating the content of information [37], communication is essential.

In nuclear power plants, with crews responding to a simulated crisis event, the best performing teams showed specific communicative characteristics: interaction patterns involving fewer team members, and using short phrases instead of complex interactions [42]. Moreover, in a study of teams working on a collaborative task in a time pressure situation, corrections in speech and closed loop communication increased their shared understanding and knowledge [35].

#### Shared understanding and knowledge

Teams with the opportunity to get acquainted with each other in preceding trainings, showed better performance, more efficiency and more resilience in a stressful simulated training [45]. Particular knowledge about team members, concerning their expertise, skills, abilities and preferences seemed useful. This is captured in the term shared mental model (SMM) defined as ‘a shared organized understanding and mental representation of key elements of the teams’ relevant environment’ [44]. This understanding could be optimized by investing time in exploring possibilities to reflect on the division of roles [34]. Additionally, by sharing experiences teams may be equipped with the flexibility to adapt to a changing and unpredictable environment [34]. This need for adaptive behaviour was exhibited by leaders of nuclear power plant crews as well: their teams showed better performances in simulated training sessions than the teams with less flexible and adaptive behaviour [47].

Closely related to shared mental model is knowing what others in the team know, for which the term ‘transactive memory’ is used, defined by Austin [48], as cited in Pearsall et al. [40]: ‘a combination of knowledge possessed by each individual, and a collective awareness of where knowledge resides within the team’. Improvements in transactive memory were seen when teams dealt with challenge stressors with a problem solving coping strategy [40]. The appraisal of challenge stressors was an opportunity for growth as a team, through which team members were informed of and learned from each other’s knowledge [40].

#### Situational awareness

Working successfully in a certain environment means accurately perceiving and understanding this environment. This perception and understanding is defined as situational awareness [41]. A measure of situational awareness was whether or not team members recognized a need for information with their colleagues [38]. Teams who anticipated better on this information need performed in a more efficient way than teams with lower levels of anticipation [38, 47].

In contrast with an adequate understanding of a situation, overconfidence in situational awareness has also been described [41]. Team members could feel they are in command of a situation (subjective SA), but in fact they are not (objective SA): overconfidence in situational awareness is present. In a team of soldiers, team members with a high level of subjective SA, but low levels of objective SA could feel overconfident, resulting in an increase in risk-taking and a possible decline in team performance [41]. A feeling of team engagement within the team prevented team members from feeling overconfident, resulting in less risk-taking in a stressful simulated scenario [41]. Thus, team engagement may help in reaching adequate situational awareness.

### Training possibilities for teams

The possibility of training teams for stressful situations was discussed explicitly in three studies that focused on cross training [38, 45, 46]. Cross training is a practice where teams can experience the different roles and tasks of each member through hands-on training [46]. Two studies reported positive effects of cross training, ranging from an overall more positive affective experience on the team level in comparison to another training methodology [46], and an increase in the accuracy of shared mental models [38]. The third study, with teams of naval cadets, focused mainly on the phenomenon of team familiarity: comprehensive knowledge on individual differences in competencies, skills, and abilities within the team [45]. Both teams with and without team familiarity were trained through cross training, and it seemed that teams with team familiarity were better at identifying changes in the team and their teammates [45].

More general comments on training were made in the remaining articles, ranging from the importance of fostering the perception of team engagement [41] to training in order to promote brief team interaction patterns [42].

## Discussion

### Main findings

Through this scoping review we have learned valuable lessons on team performance in stressful situations from disciplines outside the field of healthcare. Our main goal was to deepen our understanding of the concept of stress and its effects on team performance, and strategies and potential interventions to deal with stress. Because the psychological effects of stress are generic, and scientific insights and innovative strategies are sometimes more advanced in fields outside of medicine, we feel that it is important to learn from these fields.

We found a myriad of study designs in the included articles, and many studies were multi- or mixed methods, signifying the complexity of the topic. Sources of stress found in the data could be categorized as performance pressure, role pressure and time pressure. The main effects of these stressors on teams were at a cognitive level: a narrowing of focus, diminished understanding of the situation and uncertainty in roles and responsibilities. To support or improve team performance in stressful situations, situational awareness, transactive memory and a shared mental model were considered important. Investing time in proficient communication during team performance under pressure might result in improvement of these cognitive outcomes. This is backed up by previous research about rudeness and incivility and their adverse consequences on team performance [49, 50]. An intervention investigated in several included studies is cross training. Cross training was found to increase knowledge about team members, and might lead to an improved shared mental model.

### The ‘team brain’

Three of the themes we found showed overlap, namely: situational awareness, transactive memory and shared mental model. A key resemblance is that these three revolve around the development of a common view of the situation, for which communication is important. We would like to introduce the term ‘team brain’ as an umbrella term under which these themes come together.

In previous medical research, authors have talked about team cognition as an important asset for teamwork [51–53]. Team mental models and transactive memory support a team in the adaptation to sudden changes in the condition of a patient [52]. For example, surgery residents exhibited SMM-development with other team members after participating in a stressful simulation scenario (e.g. fire in the operating room during a laparoscopic task) [54]. Simulation training might have the potential to develop these cognitive structures [54]. After being involved in a trauma simulation, professionals in the emergency department showed an increase in transactive memory, which was associated with improved team performance as well [55]. An integration of the three cognitive themes and team performance in stressful situations has not yet been specified.

Three of the included studies [38, 45, 46] investigated cross training as a useful intervention for teams to improve their teamwork in stressful situations. We are aware of only a few studies within the medical field mentioning cross training as a useful intervention [56–60]. However, the results from our included studies suggest the effectiveness of this type of training, specifically the positional rotation variant where team members act in the role of their colleagues for a short time [38, 46]. Knowledge about the roles of your team members could add to the development of a ‘team brain’.

### Leads for training and further research

Interprofessional training of ad hoc teams in healthcare has gained a lot of interest and progression over the past few years [19, 61–63]. These trainings often focus on behaviour, aiming to follow a certain algorithm for a specific clinical scenario like CPR. Our review suggests that for an affective process like stress, shared cognition of a team could also be a valuable focus of training. Development of a team brain, consisting of a shared mental model, situational awareness and transactive memory, could lead to an increase in team performance in stressful situations and subsequently improve patient outcomes [64, 65].

The diverse use of concepts like shared mental model, situational awareness and transactive memory calls for a clear definition that can be used to implement them in training methodologies. A recent review on shared mental models [59] confirms that a clear definition of this important cognitive outcome is still lacking. Floren et al. argued that consistent use of a definition is necessary to design and evaluate interventions that can improve collaboration of healthcare professionals [59]. Our research does not specifically address this point, but does emphasize the importance of a team brain when working under stressful circumstances. Future studies could focus on further clarification of this concept and how it can positively affect team performance of medical teams. Interventions that might contribute to the development of a team brain in an ad hoc team are possible further areas for future research. Reid et al. proposed a “Zero Point Survey” to further improve safety and performance during resuscitation [66]. They showed that an extra readiness check of self, team and environment, right before the primary survey of the patient, followed by an update and priorities, could help in developing a shared mental model. Interesting interventions like these warrant further research.

The different studies on cross training included in this review have shown the potential positive effect of this training methodology. However, by quickly scanning the literature on healthcare teams and cross training, we found a lack of studies specifically focusing on the effect of cross training in ad hoc teams working in stressful conditions. This might be an interesting topic for future research, specifically the role of cross training in the development of a ‘team brain’.

### Limitations

We found a small number of articles eligible for our review. Our initial search yielded many studies examining teamwork, but study outcomes often were described at an individual rather than team level or did not consider the effects of stress. Therefore, our strict eligibility criteria may have resulted in a restricted number of included studies on the concepts of stress and team performance. However, it does provide us with the insight that this topic requires more thorough research, which is in line with Groombridge et al. who studied decision-making during a potential stressful situation like CPR [67].

Although a thorough search was conducted, there is a possibility we missed articles on the topic. Our search was restricted to three databases (PsycINFO, Web of Science and Scopus), and to English articles only. Nonetheless, we believe the studies included in our review shed a different light on the topic, especially since our search has come up with “out of the box” journals.

We did not include studies from medical professions, and there was a lot of diversity in quality and design of the included studies. This could be interpreted as a lack of rigour. However, our novel way of looking at team performance under stress aimed to unlock potential new insights, and to learn from other disciplines. We believe our results achieved to do so. Moreover, we think the diversity of study designs implies that the study of stress and team performance is complex, multi-faceted, and warrants further research. Finally, the diversity of backgrounds within the interprofessional research team could be worthwhile and provide the reader with a different view.

## Conclusion

Critical patients demand the highest level of care by emergency teams. At the same time, stress can decrease team performance dramatically. By looking beyond the discipline of healthcare, we gain valuable insights in the effect of stress on team performance. Stress can be caused by performance pressure, role pressure and time pressure and has negative effects on focus, clear vision of responsibilities and an understanding of the situation. We have shown that detrimental effects of stress on team performance may be mitigated through development of a ‘team brain’. Such a ‘team brain’ could encompass situational awareness, transactive memory and shared mental model, which are necessary for excellent team performance in stressful situations. Communication connects these themes within the team. Further research into the development and use of this ‘team brain’ in stressful situations is needed to be able to integrate the concept in training and education, to create the high-performance team our patients need.

## Supplementary Information

**Additional file 1.**

**Additional file 2.**

**Additional file 3.**

## Data Availability

All data generated or analysed during this study are included in this published article (and its supplementary information files).

## Abbreviations

CPR: Cardiopulmonary resuscitation
CRM: Crew resource management
PRISMA: Preferred reporting items for systematic reviews and meta-analyses
SA: Situational awareness
PA: Positive affect
SMM: Shared mental model
