# Supplementary material for: Learning about stress from building, drilling and flying: a scoping review on team performance and stress in non-medical fields
Source: Scand J Trauma Resusc Emerg Med. 2021 Mar 25;29:52. doi: 10.1186/s13049-021-00865-7 (PMC7993475; doi:10.1186/s13049-021-00865-7)
Supplement: Supplementary file 1 — Additional file 1. [file 13049_2021_865_MOESM1_ESM.docx]

SUPPLEMENTARY FILE 1

### Scopus History October 27, 2020

| **Search** | **Scopus Query – October 27, 2020** | **Items found** |
| --- | --- | --- |
| #9 | (((TITLE-ABS-KEY ( team* OR crew OR aircrew ) OR TITLE-ABS-KEY ( ( cockpit OR pilots OR "air traffic" OR airforce OR "air force" OR aviation OR aviator OR army OR "armed forces" OR military OR soldier* OR seafare* OR navy OR naval OR maritim* OR police OR "law enforcement" OR "mine work*" OR minework* OR "operating room*" OR "oil industr*" OR "high reliability industr*" OR "high reliability organi*" OR "high stakes organi*" OR "hazardous industr*" OR sport* OR fire* OR nasa OR "rescue work*" OR astronaut* ) AND ( collaborat* OR cooperati* ) )) AND (TITLE-ABS-KEY ( stress* OR anxiety OR distress* OR threat OR pressure )) AND (TITLE-ABS-KEY ( coping OR cope OR deal* OR mechanism* OR attitude OR management OR managing OR adapt* OR resilience )) AND (TITLE-ABS-KEY ( fail* OR success* OR outcome OR performanc* OR improv* OR develop* OR deteriorati* OR impact OR effect* ))) AND NOT (TITLE-ABS-KEY ( patient* ))) AND (PUBYEAR > 2007) AND LANGUAGE (english) AND DOCTYPE(ar OR re OR ip) (((TITLE-ABS-KEY ( team* OR crew OR aircrew ) OR TITLE-ABS-KEY ( ( cockpit OR pilots OR "air traffic" OR airforce OR "air force" OR aviation OR aviator OR army OR "armed forces" OR military OR soldier* OR seafare* OR navy OR naval OR maritim* OR police OR "law enforcement" OR "mine work*" OR minework* OR "operating room*" OR "oil industr*" OR "high reliability industr*" OR "high reliability organi*" OR "high stakes organi*" OR "hazardous industr*" OR sport* OR fire* OR nasa OR "rescue work*" OR astronaut* ) AND ( collaborat* OR cooperati* ) )) AND (TITLE-ABS-KEY ( stress* OR anxiety OR distress* OR threat OR pressure )) AND (TITLE-ABS-KEY ( coping OR cope OR deal* OR mechanism* OR attitude OR management OR managing OR adapt* OR resilience )) AND (TITLE-ABS-KEY ( fail* OR success* OR outcome OR performanc* OR improv* OR develop* OR deteriorati* OR impact OR effect* ))) AND NOT (TITLE-ABS-KEY ( patient* ))) AND (PUBYEAR > 2007) AND LANGUAGE (english) AND DOCTYPE(ar OR re) | 4087 |
| #8 | ( ( ( TITLE-ABS-KEY ( team* OR crew OR aircrew ) OR TITLE-ABS-KEY ( ( cockpit OR pilots OR "air traffic" OR airforce OR "air force" OR aviation OR aviator OR army OR "armed forces" OR military OR soldier* OR seafare* OR navy OR naval OR maritim* OR police OR "law enforcement" OR "mine work*" OR minework* OR "operating room*" OR "oil industr*" OR "high reliability industr*" OR "high reliability organi*" OR "high stakes organi*" OR "hazardous industr*" OR sport* OR fire* OR nasa OR "rescue work*" OR astronaut* ) AND ( collaborat* OR cooperati* ) ) ) AND ( TITLE-ABS-KEY ( stress* OR anxiety OR distress* OR threat OR pressure ) ) AND ( TITLE-ABS-KEY ( coping OR cope OR deal* OR mechanism* OR attitude OR management OR managing OR adapt* OR resilience ) ) AND ( TITLE-ABS-KEY ( fail* OR success* OR outcome OR performanc* OR improv* OR develop* OR deteriorati* OR impact OR effect* ) ) ) AND NOT ( TITLE-ABS-KEY ( patient* ) ) ) AND ( PUBYEAR > 2008 )( ( ( TITLE-ABS-KEY ( team* OR crew OR aircrew ) OR TITLE-ABS-KEY ( ( cockpit OR pilots OR "air traffic" OR airforce OR "air force" OR aviation OR aviator OR army OR "armed forces" OR military OR soldier* OR seafare* OR navy OR naval OR maritim* OR police OR "law enforcement" OR "mine work*" OR minework* OR "operating room*" OR "oil industr*" OR "high reliability industr*" OR "high reliability organi*" OR "high stakes organi*" OR "hazardous industr*" OR sport* OR fire* OR nasa OR "rescue work*" OR astronaut* ) AND ( collaborat* OR cooperati* ) ) ) AND ( TITLE-ABS-KEY ( stress* OR anxiety OR distress* OR threat OR pressure ) ) AND ( TITLE-ABS-KEY ( coping OR cope OR deal* OR mechanism* OR attitude OR management OR managing OR adapt* OR resilience ) ) AND ( TITLE-ABS-KEY ( fail* OR success* OR outcome OR performanc* OR improv* OR develop* OR deteriorati* OR impact OR effect* ) ) ) AND NOT ( TITLE-ABS-KEY ( patient* ) ) ) AND ( PUBYEAR > 2007 ) | 7153 |
| #7 | #5 AND NOT #6 | 10408 |
| #6 | TITLE-ABS-KEY ( patient* ) | 8886397 |
| #5 | #1 AND #2 AND #3 AND #4 | 20071 |
| #4 | TITLE-ABS-KEY ( fail* OR success* OR outcome OR performanc* OR improv* OR develop* OR deteriorati* OR impact OR effect* ) | 37909835 |
| #3 | TITLE-ABS-KEY ( coping OR cope OR deal* OR mechanism* OR attitude OR management OR managing OR adapt* OR resilience ) | 12436064 |
| #2 | TITLE-ABS-KEY ( stress* OR anxiety OR distress* OR threat OR pressure ) | 6400714 |
| #1 | TITLE-ABS-KEY ( team* OR crew OR aircrew ) OR TITLE-ABS-KEY ( ( cockpit OR pilots OR "air traffic" OR airforce OR "air force" OR aviation OR aviator OR army OR "armed forces" OR military OR soldier* OR seafare* OR navy OR naval OR maritim* OR police OR "law enforcement" OR "mine work*" OR minework* OR "operating room*" OR "oil industr*" OR "high reliability industr*" OR "high reliability organi*" OR "high stakes organi*" OR "hazardous industr*" OR sport* OR fire* OR nasa OR "rescue work*" OR astronaut* ) AND ( collaborat* OR cooperati* ) ) | 581582 |

### APA PsycInfo Search History October 27, 2020

| **Search** | **APA – PsycInfo - Query October 27, 2020** | **Items found** |
| --- | --- | --- |
| S8 | S7 Limiters - Publication Year: 2008-2021 - Narrow by Language: - English | 2136 |
| S7 | S6 AND S3 AND S4 AND S5 | 3255 |
| S6 | S1 OR S2 | 85761 |
| S5 | DE ("Performance" OR "Group Performance" OR "Job Performance" OR "Motor Performance") OR TI (performance* OR fail* OR success* OR outcome OR performanc* OR improv* OR develop* OR deteriorati* OR impact OR effect*) OR AB (performance* OR fail* OR success* OR outcome OR performanc* OR improv* OR develop* OR deteriorati* OR impact OR effect*) OR KW (performance* OR fail* OR success* OR outcome OR performanc* OR improv* OR develop* OR deteriorati* OR impact OR effect*) | 2922125 |
| S4 | DE ("Coping Behavior" OR "Resilience (Psychological)" OR "Adaptability (Personality)" OR "Attitudes") OR TI (coping OR cope OR deal* OR mechanism* OR attitude OR managing OR management OR resilience* OR adapt*) OR AB (coping OR cope OR deal* OR mechanism* OR attitude OR managing OR management OR resilience* OR adapt*) OR KW (coping OR cope OR deal* OR mechanism* OR attitude OR managing OR management OR resilience* OR adapt*) | 1029168 |
| S3 | DE ("Occupational Stress" OR "Physiological Stress" OR "Psychological Stress" OR "Social Stress" OR "Stress Reactions" OR "Distress" OR "Anxiety" OR "Performance Anxiety" OR "Test Anxiety" OR "Stress" OR "Threat") OR TI (stress* OR anxiety OR distress* OR pressure OR threat) OR AB (stress* OR anxiety OR distress* OR pressure OR threat) OR KW (stress* OR anxiety OR distress* OR pressure OR threat) | 586747 |
| S2 | ((DE ("Aerospace Personnel" OR "Aircraft Pilots" OR "Astronauts" OR "Athletes" OR "Sports" OR "Fire Fighters" OR "Rescue Workers" OR "Police Personnel" OR "Law Enforcement Personnel" OR "Military Personnel" OR "Air Force Personnel" OR "Army Personnel" OR "Marine Personnel" OR "Military Medical Personnel" OR "Navy Personnel") OR TI (cockpit OR pilots OR “air traffic” OR airforce OR “air force” OR aviation OR aviator OR army OR “armed forces” OR military OR soldier* OR seafare* OR navy OR naval OR maritim* OR police OR “law enforcement” OR “mine work*” OR minework* OR “operating room*” OR “oil industr*” OR “high reliability industr*” OR “high reliability organi*” OR “high stakes organi*” OR “hazardous industr*” OR sport* OR fire* OR nasa OR “rescue work*” OR astronaut*) OR AB (cockpit OR pilots OR “air traffic” OR airforce OR “air force” OR aviation OR aviator OR army OR “armed forces” OR military OR soldier* OR seafare* OR navy OR naval OR maritim* OR police OR “law enforcement” OR “mine work*” OR minework* OR “operating room*” OR “oil industr*” OR “high reliability industr*” OR “high reliability organi*” OR “hazardous industr*” OR sport* OR fire* OR nasa OR “rescue work*” OR astronaut*) OR KW(cockpit OR pilots OR “air traffic” OR airforce OR “air force” OR aviation OR aviator OR army OR “armed forces” OR military OR soldier* OR seafare* OR navy OR naval OR maritim* OR police OR “law enforcement” OR “mine work*” OR minework* OR “operating room*” OR “oil industr*” OR “high reliability industr*” OR “high reliability organi*” OR “hazardous industr*” OR sport* OR fire* OR nasa OR “rescue work*” OR astronaut*)) AND (DE ("Collaboration" OR "Cooperation") OR TI (collaborat* OR cooperati*) OR AB (collaborat* OR cooperati*) OR KW (collaborat* OR cooperati*))) | 5817 |
| S1 | DE ("Teams" OR "Work Teams" OR "Self-Managing Work Teams") OR TI (team* OR crew OR aircrew) OR AB (team* OR crew OR aircrew) OR KW (team* OR crew OR aircrew) | 80949 |

### Web of Science Core Collection History October 27, 2020

| **Search** | **Web of Science Core Collection – Query October 27, 2020** | **Items found** |
| --- | --- | --- |
| #7 | #5 NOT TS= (patient*)  Refined by: LANGUAGE: (English); DOCUMENT TYPES: ( Article OR Early Access OR Review)  Indexes=SCI-EXPANDED, SSCI, A&HCI, ESCI Timespan=2008-2021 | 2872 |
| #6 | #5 NOT TS= (patient*)  Indexes=SCI-EXPANDED, SSCI, A&HCI, ESCI Timespan=All years | 3840 |
| #5 | #4 AND #3 AND #2 AND #1  Indexes=SCI-EXPANDED, SSCI, A&HCI, ESCI Timespan=All years | 7422 |
| #4 | TS= (Fail* OR success* OR “outcome” OR performanc* OR improv* OR develop* OR deteriorati* OR “impact” OR effect*)  Indexes=SCI-EXPANDED, SSCI, A&HCI, ESCI Timespan=All years | 22669708 |
| #3 | TS= (“Coping” OR “cope” OR deal* OR mechanism* OR “attitude” OR “management” OR “managing” OR adapt* OR “resilience”)  Indexes=SCI-EXPANDED, SSCI, A&HCI, ESCI Timespan=All years | 7310841 |
| #2 | TS= (“stress*” OR “anxiety” OR “distress*” OR “threat” OR “pressure”)  Indexes=SCI-EXPANDED, SSCI, A&HCI, ESCI Timespan=All years | 3827317 |
| #1 | TS= (team* OR “crew” OR “aircrew”) OR TS= ((cockpit OR “pilots” OR “air traffic” OR “airforce” OR “air force” OR “aviation” OR “aviator” OR “army” OR “armed forces” OR “military” OR soldier* OR seafare* OR “navy” OR “naval” OR maritim* OR “police” OR “law enforcement” OR “mine work*” OR minework* OR “operating room*” OR “oil industr*” OR “high reliability industr*” OR “high reliability organi*” OR “high stakes organi*” OR “hazardous industr*” OR sport* OR fire* OR “nasa” OR “rescue work*“ OR astronaut*) AND (collaborat* OR cooperati*))  Indexes=SCI-EXPANDED, SSCI, A&HCI, ESCI Timespan=All years | 241738 |
