# Supplementary material for: Learning about stress from building, drilling and flying: a scoping review on team performance and stress in non-medical fields
Source: Scand J Trauma Resusc Emerg Med. 2021 Mar 25;29:52. doi: 10.1186/s13049-021-00865-7 (PMC7993475; doi:10.1186/s13049-021-00865-7)
Supplement: Supplementary file 2 — Additional file 2. [file 13049_2021_865_MOESM2_ESM.docx]

SUPPLEMENTARY FILE 2

**Overview criteria of eligibility**

**Phase 1 Screening titles**

Inclusion

- Team learning
- Coping with stressors in team
- Training program for teams
- Decision making in stress
- Characteristics of team leadership
- Sources of stress in team
- Measurement of stress in teams
- Team resilience
- Improve performance of teams

Exclusion

- Not about humans
- Not about performance of a team
- Focus on survival of patients
- Diseases related to stress like burnout/PTSD
- Stress/emotions in medical personnel
- Effects of certain enzyme levels in sports
- Effect of medication
- Focus on virtual teams, not on action teams
- Workplace bullying as stress factor
- Focus only on improvement of coaching
- Socio-economic profiles
- Improvement of dietary/nutritional status
- Safety of ecological systems
- Quality of worklife
- Mapping landscapes and other geographical information
- Effect of field status in football
- Role of parents in schools
- Safety of borders
- Safety of software
- Organization of crisis management
- Consequences of trauma
- Tourism development
- Stress test of surveillance systems
- Health care teams
- Work-related stress
- Pregnancy-related problems
- Pressure on skin (decubitis etc)
- Risk related to (political) violence
- Effect of certain physical parameters on sports’ performance
- Stress in librarians

**Phase 2 Screening abstracts**

Step 1 (article has to meet all 3 criteria)

- Teams working together (with a shared goal)
- In real-life (not virtual)
- In stressful situations, or under pressure (time pressure, performance as pressure)

Step 2 (one or more of the criteria below should be met)

- Stress: cognitive appraisal and coping
- Influence of stress on teamwork or team performance (specific aspects: communication, collaboration, cooperation, shared mental model, team leadership)
- Interventions on coping with stressful situations (with goal to improve team performance)

**Phase 3 Screening full-text**

Inclusion

- Teams working together (with a shared goal), in real-life and under pressure (time pressure, performance as a pressure)
- Ideas on cognitive appraisal and coping with stress
- Influence of stress on teamwork or team performance (specific aspects: communication, collaboration, cooperation, shared mental model, team leadership)
- Interventions on coping with stressful situations (with goal to improve team performance)
- Focus on individuals with a shared goal, and with team performance as an outcome measure

Exclusion

- Description of a planned intervention/procedure/education for teams
- Detailed analysis of an incident with a team (e.g. Deepwater Horizon)
- A “How to work with teams in general”-guide (overview-articles, presentation of a framework, nothing on what exactly is going on in the teamprocess)
- “How does stress in teams work in general” – theoretical framework, what do we know about stress and teams?
- Outcome measure: nothing related to team performance or team processes
- Exclusive focus on individuals within the team > how do you deal with stress?
- Not about teamwork and / or stress
