# Supplementary material for: Learning about stress from building, drilling and flying: a scoping review on team performance and stress in non-medical fields
Source: Scand J Trauma Resusc Emerg Med. 2021 Mar 25;29:52. doi: 10.1186/s13049-021-00865-7 (PMC7993475; doi:10.1186/s13049-021-00865-7)
Supplement: Supplementary file 3 — Additional file 3. [file 13049_2021_865_MOESM3_ESM.docx]

SUPPLEMENTARY FILE 3

Format data charting form

| **General characteristics** |  |
| --- | --- |
| Title |  |
| Author |  |
| Year of publication |  |
| Journal |  |
| Research design |  |
| Country |  |
| Discipline |  |
| Objective |  |
| Hypothesis |  |
| Who is in the team and what is their task? |  |
| How is teamwork described? (Including theoretical framework, models etcetera) |  |
| Defining team performance |  |
| What is stress/pressure? (Theoretical framework/models) |  |
| Characteristics of a stressful situation |  |
| Methods/measurement |  |
| Outcomes + details |  |
|  |  |
| **Research questions** |  |
| Performance in stressful situations |  |
| Interventions/strategies to maintain good team performance |  |
| Effects of stress on team processes (or the other way around: effect of team processes on the experience of stress) |  |
| Interesting items for the future/healthcare |  |
